# Acceptance and accessibility to the early phase COVID-19 vaccination among the healthcare workers and hill tribe population in Thailand

**DOI:** 10.1038/s41598-022-15149-y

**Published:** 2022-06-30

**Authors:** Pilasinee Wongnuch, Thanatchaporn Mulikaburt, Tawatchai Apidechkul, Peeradone Srichan, Ratipark Tamornpark, Anusorn Udplong, Soontaree Suratana, Siwarak Kitchanapaibul

**Affiliations:** 1 School of Health Sciences, Mae Fah Luang University, Chiang Rai, Thailand; 2 Center of Excellence for Hill Tribe Health Research, Mae Fah Luang University, Chiang Rai, Thailand

**Keywords:** Diseases, Health care

## Abstract

Coronavirus disease 2019 (COVID-19) is a serious emerging disease and an extreme threat to human life. This study aimed to understand the perceptions of hill tribe people living in the border areas of Thailand-Myanmar and health workers regarding the acceptability and accessibility of the COVID-19 vaccine and health workers’ perceptions of the readiness to implement the vaccination program during the early period of national COVID-19 vaccination. A qualitative method was applied to elicit information from key informants who lived in hill tribe villages and the health professionals who served them. The study was conducted in seven hill tribe villages located along the Thailand-Myanmar borders in Mae Fah Luang District, Chiang Rai Province, Thailand. The participants were hill tribe villagers aged 20 years and over; public health care professionals working in village health centers who had primary roles in implementing disease prevention and control measures; and public health care professionals working in districts and provincial public health offices who had primary roles in policy development and implementation. A total of 63 participants (26 men and 37 women) from seven hill tribe villages provided information. Three acceptance choices regarding receiving the COVID-19 vaccine were found among the hill tribes: definite acceptance, likely acceptance, and no preference. Two factors related to obtaining access to the new COVID-19 vaccine were found: Thai citizenship and the level of literacy related to the vaccine. There was no process or protocol in place for implementing the new vaccine among health professionals working at the district, subdistrict, or community levels, but the national expanded immunization program (EPI) system was clearly demonstrated to extend throughout the health service chain in Thailand. During the early period of national COVID-19 vaccine implantation in Thailand, not all members of the hill tribes accepted the vaccine; participant acceptance depended on several factors, including a participant’s previous experience with vaccination, whether he or she required more information before making a decision, etc. While acceptance of the vaccine depended on the individual’s background, not everyone had an equal opportunity to access the vaccine. The new COVID-19 vaccine should be available at the village level, including in hill tribe villages, to reduce the systemic threat to the country.

## Introduction

Coronavirus disease 2019 (COVID-19) emerged in late 2019 in China^1^ before spreading to become a new pandemic. The disease has impacted all people around the world^2–4^, regardless of whether they live in urban or rural areas^5,6^. Humans are the target of infection, and the disease has killed over one million individuals in the past few months^7^; there is no exact estimate of the number of possible deaths in the future. Particularly among those living in developing countries, many dual health burdens from the pandemic have clearly been present, such as viral hepatitis care in Africa^8^ and the dual burden of Zika and COVID-19 in India^9^. Currently, many public health interventions have been developed by various organizations^10^. Some of the interventions have proven to be successful and able to save lives^11^, while many others have not^12^. Many interventions have involved the actions of the state, government^13^ and local people^14–16^. As a result, many of these interventions have been developed far from actual people, particularly those living long distances from cities and those staying safe in their personal spaces^17^. Finally, many effective brands of vaccines have been developed to save lives^18^, and countries (including Thailand) are preparing to implement them to safeguard their citizens^19^.

Thailand is defined as a upper-middle-income country^20^, and more than 70 million people live in the country^21^. The World Health Organization (WHO) recognizes Thailand as having one of the best health systems, particularly in improving access to care through a universal health coverage scheme that aims to improve the accessibility of health care services for the Thai population^22^. However, universal coverage does not cover those who are not granted Thai identification (ID) cards, which display the cardholder’s 13-digit ID number and are used to access all public services in Thailand^23^. Immunization is one of the critical missions of health systems and health care professionals under the Ministry of Public Health, Thailand^24^. The Ministry of Public Health launched an expanded immunization program (EPI) to promote immunization to all targeted populations, particularly to prevent childhood diseases, years ago^25–27^. The program has been incorporated into services provided by all health institutes around the country so that all Thai people will be able to access the service^27^. The ultimate goal of the service is for it to be highly accessible by all people^28^. Thus, in the past few years, the Ministry of Public Health has had many experiences in providing services, particularly vaccine implementation, including the implementation of flu vaccines, to vulnerable populations throughout the country^29,30^.

Most of the actual implementation of these programs has been mediated by health care professionals who are working at public health institutes. The EPI is provided mainly to those who have Thai ID cards^23^. However, people who do not hold Thai citizenship might not be able to access services; these people include members of the hill tribes and stateless populations living in border areas. In the general health system in Thailand, clients or patients who hold Thai citizenship are required to first access health services at a hospital close to their home. If more expert care is needed, clients or patients will be referred to a higher-level hospital to meet a specialist^31^.

The hill tribe people have moved down from southern China over a couple of centuries and settled in northern Thailand, especially at the border of Thailand and Myanmar^32^. The hill tribes have their own language, culture and lifestyle, which differ completely from those of other Thais^32^. The hill tribes grow traditional subsistence crops and perform their own religious rituals^33,34^. A very small proportion of those aged over 45 years have ever attended a school. There are six main groups: Akha et al.^35^. In 2020, approximately 4.5 million individuals from the hill tribes lived in Thailand^36^.

More than 30% of hill tribe people have not been granted a Thai ID card, which is used to assess all public services, including vaccinations^37–39^; consequently, these people will not be able to access the COVID-19 vaccine on the day of its implementation^40^. The COVID-19 vaccine, which is a new vaccine, is not guaranteed to be accessible by all people. Experiences with flu vaccine implementation, health literacy level and other factors were found to be the major critical barriers to achieving the goals of vaccine implementation^29,37^. Most of the reported COVID-19 cases were from central Thailand, while few cases were reported among the hill tribe populations living in border areas; therefore, these populations might not be prioritized as the first to receive the vaccine in Thailand^41^. At the time when the study was conducted, only Sinovac^®^ was available for Thai people living in the main cities and mainly managed by a doctor. Therefore, this study aimed to understand the perceptions of the hill tribe people living in the border areas of Thailand-Myanmar and health workers regarding the acceptability and accessibility of the COVID-19 vaccine and health workers’ perceptions of the readiness to implement the vaccination program during the early period of national COVID-19 vaccination.

## Methods

**Figure 1:**
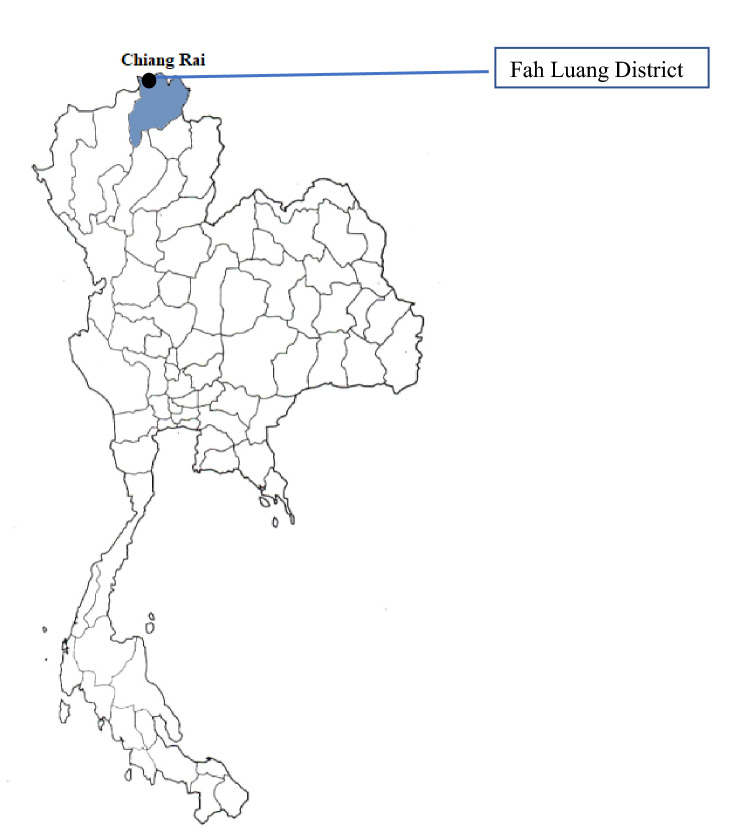
Map of the study hill tribe villages in Mae Fah Luang District, Ching Rai Province, Thailand.

A qualitative approach was applied to gather the information from the selected participants. The participants were purposively selected from seven hill tribe villages located along the Thailand-Myanmar borders in Mae Fah Luang District, Chiang Rai Province (Fig. 1). The key informants were hill tribe villagers aged 20 years and over, public health care professionals who were working in village health centers, and public health care professionals who were working in districts and provincial public health offices.

Interview guides were developed by the research team. The guides had not been used previously. Finally, three main issues were included in the set of questionnaires: acceptance, accessibility, and readiness. Concerning acceptance, five questions were used to gather the following information: (a) If the COVID-19 vaccine is available, do you plan to get the vaccine? (b) How do you think the COVID-19 vaccine is able to protect against the disease? (c) Do you worry about getting the COVID-19 vaccine? (d) What do you expect from getting a vaccine? (e) How do you plan to be involved in COVID-19 implementation in your community?

Six questions were used to collect information on the accessibility of a COVID-19 vaccine: (a) Is there any possibility of your getting the vaccine? (b) Would you bring your family members to get a vaccine? Why? (c) Do you have any barriers to getting the vaccine? Why? (d) If you are not among the first group of Thais to get a vaccine, what would you think about that? (e) How difficult or easy is it for you to access a hospital for vaccination? (f) Could you please clarify the information regarding the COVID-19 vaccine?

Five questions were used to gather information on readiness to implement the COVID-19 vaccine: (a) What do you think about the policy for implementing the COVID-19 vaccine? (b) Have you been trained in administering the COVID-19 vaccine? (c) How did you design the system for COVID-19 vaccine implementation? (d) Do you have any other resources to support the implementation of the COVID-19 vaccine? Please clarify. (e) How can the vaccine address the problem? (Additional File 1: Question guide).

The quality of the interview guides was evaluated by a medical doctor, a community nurse, and public health care workers who were working in the immunization programs. The interview guides were also piloted among five selected hill tribe people and one health care professional. Afterward, the questions were revised again by the research team before use in the field. The sets of questions on acceptance and accessibility were used among both the hill tribe villagers and health care professionals. The set of questions on readiness was used among the health care professionals and public health volunteers in the villages.

All research team members sat down together and confirmed their understanding of the context of the questions before the project commenced. The information was collected from seven villages, three health-promoting hospitals, one district public health office, and another provincial public health office. Public health officers who were directly involved in COVID-19 prevention and control, including vaccine implementation, were selected. Before the information was collected, all selected village heads were contacted and provided with essential information, such as the study objective, protocols, the rights of participants, and the use of the obtained information. The characteristics of the key informants were discussed, and the informants were then selected. The main participants were village leaders who were working as village heads; village health volunteers; and members of the local administration who were elected by village members, elderly individuals, and other working age populations.

Hill tribe participants were selected by a purposeful method by those who belonged to a hill tribe and spoke Thai fluently. Participants who were health care professionals were selected based on the relationship of their job to COVID-19 prevention and control. Hill tribe participants were asked questions on two aspects, namely, acceptance and accessibility, while the health care professionals were asked questions on all aspects. All selected key informants were informed in advance by the village heads. On the date of data collection, selected participants were provided with all essential information. Informed consent was obtained in writing or by fingerprinting before starting the interview in a confidential room at the participants’ homes. For participants who could not speak Thai, a community health volunteer was asked to help gather the information. Data were collected during the second to fourth week of January 2021. The interviews were conducted by three researchers (one medical anthropologist, one medical behavioral expert, and one public health expert) who had strong experience and knowledge in qualitative research methods. All interviews were audio-recorded after obtaining approval from the participants.

All audio-recorded interviews were transcribed in Thai and double checked by reading the transcript while simultaneously listening to the audio recording. Before the analysis was performed, all typed information was sent to the participants, who were asked to verify the correctness of the information. Data were entered into the NVivo program (NVivo, qualitative data analysis software; QSR International Pty Ltd., version 11, 2015) for thematic analysis. The data analysis was conducted in parallel with the analysis by the research team, whose members had a variety of backgrounds: medical anthropology, health behavior, community health, and epidemiology. In the coding step, the concept-driven coding or approach was used. The research team read the transcriptions and highlighted meaningful units; coded and recoded the data and looked for abstractions in the participants’ accounts; and organized, compared, contrasted and defined the key themes according to the three aspects. Once the analysis was complete, an external expert with a background in social epidemiology was invited to audit the findings.

### Ethics approval and consent to participate

All research tools and procedures were approved by the Ethics Research Committee of the Chiang Rai Provincial Health Office, Thailand (CRPPHO No. 74/2563). All participants were informed of the details of the study and the process of consent, identification, privacy protection, and confidentiality. Participants were informed of their rights during voluntary participation and were free to withdraw at any time during the process. Confidentiality was guaranteed by an oral consent process to protect the participants’ personal information. Participants were informed that their personal information would not be used in the published data. All methods were carried out in accordance with relevant guidelines and regulations. Both the electronic files and the hard copies of the data were destroyed after the conclusions were drawn.

## Results

A total of 63 people from seven villages (4–10 persons from each village) participated in the study. The participants comprised 26 men and 37 women. The average age of the participants was 49.3 years (min = 20, max = 90). The general characteristics of the participants were as follows: Twenty-eight participants were Thai-Yai, fourteen were Yunnan Chinese, eight were Akha, nine belonged to other minor tribes, and five were health care professionals. In addition, fifty-two participants were Buddhist, and eleven were Christian; thirty were illiterate, and thirty-three had attended a school; and forty were unemployed, eighteen were working on a farm, and five were health care professionals (Table 1).

**Table 1:** Demographics (n = 63).

Characteristic	n	%
Total	63	100.0
**Gender**		
Male	26	41.3
Female	37	58.7
**Age (years)**		
20–30	11	17.5
31–40	14	22.2
41–50	11	17.5
51–60	9	14.3
61 and over	18	28.6
Mean age = 49.3, Min = 20, Max = 90		
**Marital status**		
Single	9	14.3
Married	41	65.1
Ever married	13	20.6
**Education**		
Illiterate	30	47.6
Primary school	8	12.7
High school	20	31.7
University	5	8.0
**Tribe**		
Tai Yai	28	44.4
Yunnan-Chinese	14	22.2
Akha	8	12.7
Thai	5	8.0
Lisu	3	4.8
Lahu	4	6.3
Yao	1	1.6
**Religion**		
Buddhist	52	82.5
Christian	11	17.5
**Occupation**		
Unemployed	40	63.5
Agriculture	18	28.5
Healthcare worker	5	8.0
Health-promoting hospital	3	60.0
District and provincial public health office	2	40.0

Among the participants who were health care professionals, three worked at health institutes in village settings, and their primary roles were disease prevention and control. Two worked at district and provincial public health offices, and their primary roles were policy development and implementation. Four were males, and one was female. Three were public health workers, and two were nurses. The average age was 36 years, and all five had graduated university (Table 1).

Three major aspects were presented in this study: acceptance, accessibility, and readiness of COVID-19 vaccine implementation among hill tribe people.

### Acceptance

Less than half of the participants clearly knew the definition and advantages of the COVID-19 vaccine. Moreover, only eleven (11) participants knew about the availability of the COVID-19 vaccine. Those who were older knew less about the issue.

Concerning the acceptance of taking the COVID-19 vaccine when it became available, three categories were classified: definite acceptance, likely acceptance after receiving additional information, and no preference about accepting the vaccine.

### Definite acceptance

Those who were coded as “definite acceptance” and “likely acceptance after receiving additional information” were predominantly among the younger age groups.

A 29-year-old woman (P#33, village health volunteer) said the following:

I have been working as a village health volunteer for a while, and I think I would get a COVID-19 vaccine; it would be good for me. I will definitely get the vaccine if it is available.

A 48-year-old man (P#49, villager) indicated the following:

I agreed to provide vaccines to everyone in this area because we are living in a high-risk area. Many Burmese come across the border in our village to buy stuff every day. Fewer people came during the epidemic. Surely, I will get the COVID-19 vaccine if possible.

Moreover, the acceptance of the COVID-19 vaccine depended on the experience of the participants. Older people who had received a flu vaccine in the past were more interested in receiving the COVID-19 vaccine and were happy to say “yes” to receiving it.

A 43-year-old woman (P#25, village health volunteer) said the following:

I had the flu vaccine at Terd Thai Hospital last year with no side effects. During the rest of the whole year, I did not have the flu. I think it is a good option to have the COVID-19 vaccine. It will possibly save our life. I will bring everyone in my family to get a vaccine if it is available.

Other people reported that they would consider obtaining the COVID-19 vaccine because they did not have negative side effects after receiving another vaccine.

A 45-year-old woman (P#09, villager) said the following:

I never have side effect from vaccination in my life, then I will get COVID-19. I think it is good to me and to all people in the village.

A 33-year-old man (P#53, village health volunteer) stated the following:

I never have a problem in receiving a vaccine; I will go and get a COVID-19 vaccine when it is available.

### Likely acceptance after receiving additional information

Information from social media impacted the acceptance of the vaccine, particularly among those who were in the younger age groups. Those who were younger were able to speak Thai and were able to use and access social media through several applications, such as Facebook and Line. Facebook and Line are the two dominant social media platforms in Thailand. The hill tribe people who were fluent in Thai could receive the information quickly, regardless of whether the information was correct. Before receiving vaccines, some people needed more information, especially concerning the severe side effects or adverse effects of the COVID-19 vaccine.

A 29-year-old woman (P#33, village health volunteer) said the following:

I heard that many people died from having COVID-19 in the Netherlands in the past days on Facebook. I think I need more information before making a decision to receive the COVID-19 vaccine.

A 42-year-old man (P#31, villager) stated the following:

I will ask for more information about the vaccine before deciding to join the project. I think we need clear information. I do not follow the advice of politicians; rather, I will hear the information from the medical scientists.

### No preference for the vaccine

Many older people reported that they might prefer not receiving the vaccine and were concerned about experiencing severe side effects, and because of the limited availability of the vaccine, many older people reported that young people should receive the vaccine. The reasons for providing a vaccine to young people were that young people could support the family financially and that young people have a stronger ability to tolerate side effects.

A 78-year-old man (P#51, villager) said the following:

I think I will not get the COVID-19 vaccine because my health is currently not good. I am now practicing many things to safeguard my health, such as taking our tribal herbs and using them for daily showers. I think it could protect me from the infection. Moreover, personally, I have very few opportunities to join people outside family members. I heard that people said that avoiding people will protect against the disease.

A 64-year-old woman (P#15, villager) noted the following:

I am too old to get the vaccine. I think the young people should be vaccinated. My son has three kids; he should get the vaccine. I will ask him to get the vaccine…. without him, our family would not survive.

A 33-year-old man (P#53, village health volunteer) said the following:

I got a shot of vaccine last year at the hospital; immediately, I felt not good. I could not breathe. I was observed by a team of medical doctors. I think I will not consider receiving the COVID-19 vaccine. I am afraid my body will not react well to having a vaccine.

A 27-year-old man (P#59, nurse) said the following:

I have been working as a nurse at this hospital for five years. I think I will not join in the COVID-19 vaccine administration program. Even though I face many patients every day, as I know about the vaccine information right now, I did not join the COVID-19 program because there are many side effects and unclear points.

While some health professionals did not accept the vaccine, some other health professionals expressed their interest in joining the vaccine program.

A 27-year-old community nurse (P#59, nurse) said:

I am not planning to get a COVID-19 vaccine. I think it’s necessary to clearly prove its safety before implementation.

A 30-year health professional (P#48, health worker) said:

I have worked at this health-promoting hospital for ten years. I have been involved in implementing prevention and control measures for COVID-19 since the first day. I have had to work every day, no holidays, no weekends for a year. I perform local quarantine and check the health status of everyone who enters the villages. I accept that I am now at the high-risk stage. I very much need to get the vaccine if it is available.

### Accessibility

Participants highlighted how, according to the regulations of Thailand, only Thai citizens have free access to medical care, including immunization. Some hill tribe people absolutely accepted that they would not receive the COVID-19 vaccine because they did not have Thai ID cards.

A 68-year-old man (P#44, villager) said the following:

I have no Thai ID card. I will not be counted on a list for receiving the COVID-19 vaccine. I know that it is important and very interesting, but I have no better idea. A few days ago, I asked many times about getting the vaccine from our village public health volunteer. However, they told me it is not possible; the vaccine is only for Thai people.

A 40-year-old man (P#45, village health volunteer) supported the following:

I am voluntarily working as a public health volunteer in my community. However, I do not have a Thai ID card. I will not be able to get the vaccine. I know this automatically because whenever public health initiates any vaccine implementation, I am not included in the program because I do not have a Thai ID card. However, I am still very happy to volunteer for this work because I could help my family and my friends in some ways.

A 30-year-old health professional (P#48, health worker) said:

Even though I have been employed at one of the health-promoting hospitals, I did not hear about this as a government officer; therefore, I am not sure that I will be eligible to get the vaccine. I am now worried about that because my work today is to screen all people who enter this village.

### Readiness

The specific policy regarding COVID-19 implementation was deployed only by public health staff at the provincial level, but the policy was officially directed from the village, subdistrict, and district levels. However, vaccine interventions under the EPI clearly existed; these interventions included policies, guidelines, and materials. Under the national EPI, all vaccines (such as the tetanus, diphtheria, hepatitis B, and polio vaccines) were available in health-promoting hospitals and district hospitals at any time. The workflow, including how responders provide vaccines to health institutes at the district and subdistrict levels, was also clearly demonstrated.

A 27-year-old nurse (P#59, nurse) said the following:

We do have the processes and materials related to the vaccines under the EPI program for children and other targeted populations. We have set aside at least two days a month to provide vaccines to a mass targeted population from the villages. However, to date, we have not had a special program, schedule, or materials for the COVID-19 vaccine.

This nurse further stated:

Some people from the villages asked about the availability of the vaccine, but we did not have a good response for them yet. In general, whenever we implement a new program, particularly vaccine implementation, all public health staff who handle the vaccine will be invited to be trained in a specially designed program before launching the program. For the COVID-19 vaccine, if it will be implemented, I truly need to know everything regarding the vaccine to avoid mistakes and to be able to handle the side effects if any occur. We are now living slightly far away from the central hospital in the city; thus, we do not expect any severe side effects in our population while implementing the vaccine.

Some aspects of the process of flu vaccine delivery were used to recommend a better procedure for the COVID-19 vaccine, if applicable.

A 51-year-old woman public health staff member (P#60, health worker) said the following:

I am a nurse working as the focal point in the EPI program at the Chiang Rai provincial level. I have worked in this position for more than 25 years. I know that the best procedure to implement a vaccine to the people in a village is for the medical staff from the district level to be the leaders. We have experience in asking public health staff working at the subdistrict level of a health-promoting hospital, and the coverage of vaccine implementation was not too great compared to the outcome of medical staff working at the district hospital. Thus, we will ask the district hospital staff to handle the COVID-19 vaccine implementation if it is available. However, we do not have any specific policy from the Thailand Ministry of Public Health at the provincial level yet.

A 43-year-old man (P#61, district public health staff) who was a public health professional working in the district public health office said the following:

Basically, we are very familiar with implementing a vaccine to our population. If the government has a new policy for delivering the COVID-19 vaccine, I do not think we will have a significant problem. The only point that I am concerned about is the safety of the new vaccine. I am sure that we will have a number of questions regarding vaccine safety from the targeted population before smooth implementation.

## Discussion

Interviews with 58 hill tribe villagers and five health care professionals provided several insights on the acceptance, accessibility, and readiness of the policy and guidelines for implementing the new COVID-19 vaccine for hill tribe people who lived in the border area of Thailand-Myanmar during the early period of national COVID-19 vaccination. Three choices regarding COVID-19 vaccine acceptance among the hill tribes were found: definite acceptance, likely acceptance, and no preference. Those who had a higher education level and a younger age preferred to accept the vaccine, while some people had not yet decided and required more information before deciding. The elderly hill tribe population preferred to not accept the vaccine for several reasons, such as not tolerating side effects and preferring to give the limitedly available vaccine to their children, who were the people managing the family economy. Only some of the hill tribe people, namely, those holding Thai citizenship and those at high risk, would be able to access a vaccine. The policy and work procedures on the new COVID-19 vaccine are being implemented in a remote health care setting.

In this study, three preferences concerning the acceptance of the new COVID-19 vaccine were detected among hill tribe people: definite acceptance, likely acceptance, and no preference for the vaccine. Regarding the acceptance of the vaccine, there were several influencing factors, such as a prior negative experience in receiving a vaccine, lack of information about vaccine complications, and individual perception of the risk of the disease. Mehmood^42^ reported that the main reasons for accepting COVID-19 vaccines were that people did not believe in vaccine safety, effectiveness, and unclear severity of the side effects.

Many older individuals had experience with flu vaccines, with some people having experienced side effects. This group tended to refuse the COVID-19 vaccine, while other people who did not have negative experiences preferred to receive the vaccine. These findings coincide with those of a study by Favin et al.^43^, who reported that experience with side effects was defined as one of the reasons for parents not bringing their children to receive a vaccine. A study in India also reported that previous experience impacted the decision to accept the COVID-19 vaccine; the adverse effects of the vaccine were of particular concern^44^.

Information on COVID-19 vaccine safety and adverse effects or vaccine complications, especially regarding the number of deaths from using the COVID-19 vaccine abroad, impacted the decisions of hill tribe people. Mckee et al.^45^ reported that safety concerns were a critical factor in deciding on access to vaccine programs. This finding was supported by a study conducted in Europe; the study showed that concerns about the side effects of a vaccine were the main reason for not accessing a vaccine^46^. A study on a lack of access to the human papilloma virus vaccine in Thailand found that for young adult women, worrying about the adverse effects of the vaccine was the major factor in not accepting it^47^. Boonyatistan et al.^48^ reported that for almost two decades, Thailand has been making serious efforts to monitor the adverse effects of all vaccines implemented in the country.

This study found that many factors contributed to poor acceptance of the COVID-19 vaccine; these factors included prior negative experience with vaccination, fake news regarding the reversal effect of the vaccine, and personal knowledge and attitudes concerning vaccination. Information released from social media, such as Facebook and Line, was a major source of information for new vaccines. Information from vaccine experts on social media was only the information available for Thai people and was used to make decisions in all aspects related to COVID-19 prevention and control, including accepting a vaccine. Although the COVID-19 vaccine was unavailable to be taken or administered when this study was conducted, accurate information on the advantages of vaccination should be disseminated to these populations. A study on hill tribe child immunization^37^ reported that using the hill tribe language to improve knowledge among parents could increase access to immunization for hill tribe children. This finding clearly demonstrated that the improvement of people’s knowledge could increase access to a vaccine.

Some people who were working on the frontline to fight COVID-19 perceived that they were at risk of infection. This group of hill tribe people preferred to receive a vaccine. The WHO recommended that health care professionals who are working with COVID-19 patients should have first priority in accessing the COVID-19 vaccine^49^. The Ministry of Public Health in Thailand reported that health professionals will be the first priority group who will receive the COVID-19 vaccine once it is available to reduce their risk of infection^50^.

Wang et al.^51^ reported that as the largest population in the world, Southeast Asian people, particularly the healthy adult population, required the COVID-19 vaccine. Our study reported that holding Thai citizenship was associated with accessibility to the COVID-19 vaccine. The National Health Security Office (NHSO), Thailand, reported that based on the health security scheme, only those people who hold Thai citizenship will be able to access the COVID-19 vaccine because a large national budget will be required for the vaccine and the availability of the vaccine is very limited^52^. Therefore, some hill tribe people who have not been granted Thai citizenship will not be able to access the COVID-19 vaccine. However, if the vaccine supply is sufficient, everyone living in Thailand should be immunized with the vaccine, regardless of whether they have a Thai ID. In addition, there were some important challenges to poor access to healthcare services among hill tribe people, such as the difficulty of communication between clients and healthcare providers, i.e., language barriers, and distance^37^. These key challenges might impact the accessibility of the COVID-19 vaccine for hill tribe people.

Concerning the readiness of the health system to implement the COVID-19 vaccine in Thailand, hill tribe people who do not hold Thai citizenship are currently not ready for specific COVID-19 implementation due to the lack of policies, guidelines and required materials. However, the Thai health system has introduced many kinds of vaccines over more than 40 years^53^, and the COVID-19 vaccine can easily be implemented under the existing health system in Thailand. Moreover, Thailand has experienced the implementation of the flu vaccine for children as a new vaccine^54^, and new COVID-19 vaccine implementation is possible through the current health system. Vaccine nationalism is one of the critical challenges to COVID-19 vaccine implementation globally, particularly in developing countries, including Thailand, which requires the cooperation of the global community to address the problem^55^.

There were some limitations to this study. First, four elderly people could not speak Thai fluently, so the public health volunteers were asked to help in translation. However, typed information was returned to the four participants to confirm the answers before further analysis. Second, few health care workers who had directly worked with the COVID-19 vaccine were included in the study. Extending the number of health care workers with a partial role in implementing the COVID-19 vaccine might improve some information found in the study. Third, only people living in seven selected hill tribe villages were selected for the study. Selection of participants from widely studied areas might improve the context of the interpretation of the findings. Finally, since this study employed a qualitative approach, the generalization of the findings is not fully applicable.

## Conclusion

Hill tribe people who live in remote and border areas in Thailand are at specific risk of COVID-19 due to the spread of the disease from neighboring countries and people returning to villages after losing their jobs. The poor economic situation and education of these people make them particularly vulnerable to economic downturns from the lockdown and impair their ability to avoid being exposed to the disease. Some members of the hill tribes preferred not to receive the vaccine due to their past experiences and a lack of information about this new vaccine, especially regarding its safety. Regarding accessibility, even in this dire situation where the vaccine is the only hope for humankind, only some of the hill tribe people will meet certain criteria for accessing the new vaccine: being Thai citizens, being members of populations at risk (health care professionals and health volunteers), and being literate. The policy and system are not currently ready for COVID-19 vaccine implementation, but COVID-19 vaccination could easily be integrated into the routine immunization program, which is already extended to peripheral health care centers at the village level throughout Thailand. To introduce a new vaccine for addressing COVID-19, a strategic plan should be established that includes all people living in Thailand, and the involvement of community members should also significantly impact the outcome of the program in hill tribe communities. The implementation should be concerned with improving people’s knowledge and herd immunity by increasing the number of people participating in the program, regardless of holding a Thai ID card. The vaccine should also be available at small health-promoting hospitals located in hill tribe villages.

## Supplementary Information

Supplementary Information.

## Data Availability

The datasets used and/or analyzed during the current study are available from the corresponding author on reasonable request.

## Abbreviations

COVID-19: Coronavirus disease 2019
EPI: Expanded immunization program
ID: Identification
NHSO: National Health Security Office
WHO: World Health Organization
