# Supplementary material for: Acceptance and accessibility to the early phase COVID-19 vaccination among the healthcare workers and hill tribe population in Thailand
Source: Sci Rep. 2022 Jun 30;12:11035. doi: 10.1038/s41598-022-15149-y (PMC9244445; doi:10.1038/s41598-022-15149-y)
Supplement: Supplementary file 1 — Supplementary Information. [file 41598_2022_15149_MOESM1_ESM.docx]

**Questions guide**

**Acceptance issue**

a) If the COVID-19 vaccine is available, do you plan to get the vaccine?

b) How do you think the COVID-19 vaccine is able to protect against the disease?

c) Do you worry about getting the COVID-19 vaccine?

d) What do you expect from getting a vaccine?

e) How do you plan to be involved in COVID-19 implementation in your community?

**Accessibility of a COVID-19 vaccine issue**

a) Is there any possibility of your getting the vaccine?

b) Would you bring your family members to get a vaccine? Why?

c) Do you have any barriers to getting the vaccine? Why?

d) If you are not among the first group of Thais to get a vaccine, what do you think about that?

e) How difficult or easy is it for you to access a hospital for vaccination?

**Readiness to implement the COVID-19 vaccine issue**

a) What do you think about the policy for implementing the COVID-19 vaccine?

b) Have you been trained in administering the COVID-19 vaccine?

c) How did you design the system for COVID-19 vaccine implementation?

d) Do you have any other resources to support the implementation of the COVID-19 vaccine? Please clarify?

e) How can the vaccine handle the problem?
